# Physiological and Pathological Considerations for the Use of Flaxseed as a Therapeutic Dietary Strategy

**DOI:** 10.31083/j.rcm2405149

**Published:** 2023-05-18

**Authors:** Broderick C. Hirst, Elena Dibrov, Susan D. Hirst, Grant N. Pierce

**Affiliations:** ^1^Department of Physiology and Pathophysiology, Rady Faculty of Health Sciences, University of Manitoba, Winnipeg, MB R3T 2N2, Canada; ^2^Institute of Cardiovascular Sciences, Canadian Centre for Agrifood Research in Health and Medicine, Albrechtsen Research Centre, St. Boniface Hospital, Winnipeg, MB R2H 2A6, Canada

**Keywords:** disease, cardiovascular disease, clinical trials, diet, nutrition, flaxseed, linseed

## Abstract

The inclusion of flaxseed in the diet may have a great number of potential benefits for the well-being of both healthy individuals and those challenged by disease conditions as well. With an increase in the number and quality of studies focused on the physiological and pathophysiological effects of dietary flaxseed, our knowledge concerning the rationale for the inclusion of flaxseed in our diet has become more convincing and stronger. The purpose of this review is threefold. First, the review will comprehensively document the evidence supporting the value of dietary flaxseed to improve bodily health in both normal and disease conditions. Second, this review will identify the mechanisms of action responsible for these effects. Finally, this article will review practical aspects relevant to the inclusion of flaxseed in the diet. Briefly, supplementing the diet with flaxseed has beneficial effects on the treatment and/or prevention of different kinds of cardiovascular disease (hypertension, ischemic heart disease, myocardial infarcts, atherosclerosis), non-alcoholic fatty liver disease, breast cancer, bone strength, menopause, diabetes, and wound healing. Although some controversy exists on the component within flaxseed that provides these beneficial actions, it is likely that the rich content of the omega-3 fatty acid, alpha linolenic acid, is primarily responsible for the majority of these biological effects. It is concluded that the constantly expanding evidence in support of the inclusion of flaxseed in our daily diet to provide significant health benefits strongly encourages the initiation of additional work on dietary flaxseed in order to both confirm past findings as well as to further advance our knowledge regarding the important biological actions of dietary flaxseed.

## 1. Introduction

Despite the importance that pharmaceutical strategies have in the management of disease, it may be equally significant to remember that most chronic diseases have a cause that is in some cases almost entirely due to (i) an inadequate diet, or (ii) a diet lacking essential nutrients, or (iii) a diet too abundant in calories, or (iv) an unbalanced diet that is overly rich in fat or carbohydrates. The major causes of death in the world today are cardiovascular disease (CVD), cancer and diabetes and all three of these pathologies have strong behavioural components that include poor dietary habits [1, 2, 3]. For example, poor nutrition has been suggested to be responsible for ~40 to 90% of all CVD [4, 5]. Treating the cause rather than the symptoms of a disease is a logical approach to treat disease. Medicine is only prescribed and used when clinical symptoms are present. Nutritional strategies, however, can be used to slow the clinical appearance of a disease or even prevent its appearance entirely. Indeed, avoiding the cause of the disease in the first place can be an effective health strategy. Research data on the topic has even suggested that dietary behaviour modification and nutritional interventions can be a more effective therapeutic strategy than a pharmaceutical approach in some cases [2]. Nutritional changes can be put into practice at a lower individual cost and to a greater population with fewer side-effects than most drug-based therapies. Furthermore, the power of implementing nutritional strategies like the Mediterranean diet or supplementing the diet with omega-3 fatty acids can clearly justify their implementation instead of, or in concert with, pharmaceutical approaches to date [6, 7, 8, 9, 10]. In summary, it can be safely concluded that if the ingestion of foods can cause a disease or, alternatively prevent a disease from ever presenting itself, better nutrition is an eminently logical but often forgotten approach for the management of chronic disease. Furthermore, proper nutrition is also important in association with the pharmacological approach, as an adjuvant.

The only question remaining then is the kind of nutritional intervention that will be most easy to implement and most effective to prevent or treat chronic disease. Many grain crops have been touted as having significant health benefits. These include oats, wheat, buckwheat, hemp, rice and flax amongst others. However, supplementation of the diet with flaxseed may provide unusually beneficial health advantages that warrant their focused attention in this manuscript. This deserves additional attention because flaxseed is relatively uncommon in the marketplace today despite its long history of cultivation and ingestion for thousands of years [11].

## 2. What is the Composition of Flaxseed?

Flax, also known as *Linum usitatissimum*, is grown all over the world [12]. The flax plant produces small, brown seeds that can be ingested as is, ground into a powder or, after extraction of its oil, ingested in liquid or in pill form [13, 14, 15]. The seed mass is ~40% oil. Flax is one of the richest plant sources of the n-3 polyunsaturated fatty acid (PUFA)—α-linolenic acid (ALA) [15]. Plasma and tissue (brain, kidney, liver, heart, skeletal muscle and arteries) levels of fatty acids (saturated, monounsaturated and polyunsaturated) are significantly altered after induction of a diet supplemented with ground flaxseed [16]. Flaxseed also contains a rich source of the lignan secoisolariciresinol diglucoside (SDG). SDG is metabolized after ingestion by gut anaerobic microbes that convert them into enterolactone and enterodiol [17, 18].

Flaxseed has an excellent nutrient composition (Table 1). The composition of flaxseed, like any nutritional supplement or nutraceutical, will determine its ultimate biological actions. Each tablespoon of flaxseed contains about 55 calories. Flaxseed is low in net digestible carbohydrates due to the dominance of its carbohydrate composition by both soluble and insoluble fibre (95%), which renders flaxseed as a low carbohydrate food, ideal for inclusion in the diet of diabetic patients [19].

**Table 1.** **Nutrient composition of flaxseed**.

A. Grams per 10 grams of flaxseed or one tablespoon)				
Protein: 2	Carbohydrates: 3	Fat: 4	Sugar: 0.2	Fiber: 3
B. % composition of flaxseed				
Protein: 18	Carbohydrates: 29	Water: 7		
Fat: 42 (75% polyunsaturated and 25% saturated and monounsaturated fatty acids)				
Fiber: 28 (soluble: 20–40; insoluble: 60–80)				

Flaxseed also contains in lower concentrations cyclic peptides, cyanogenic glycosides, proteins and insoluble and soluble fibre. The cyanogenic glycosides have been suggested to have toxic actions [20]. However, this is highly unlikely to be a realistic threat to the body for several reasons, as discussed in detail elsewhere [15]. The phytic acid content of flaxseed has also been suggested to work as a chelating agent in the gut which may limit bioavailability of nutrients [21]. Again, others have concluded that the anti-nutritional compounds found in flaxseed have a minimal impact on the health of those ingesting flaxseed [22]. It is important to note that the content of all of these components will vary according to the cultivars considered [23]. The climate, soil conditions and other environmental factors will inevitably alter the constituents within flaxseed (Table 1), even if only slightly [23].

Overall, flaxseed is a relatively inexpensive food that should be easily obtained globally because it is grown in many countries throughout the world [12]. It has bioactive components of practical value in chronic disease conditions like CVD and other pathological conditions.

## 3. What are the Physiological and Pathophysiological Benefits of Supplementing the Diet with Flaxseed?

Over the last two decades, an ample amount of data has been generated in both animal and human models on how we can expect dietary flaxseed to act in the body. The amount of peer reviewed published studies on the topic should give the public a great deal of confidence that dietary flaxseed can produce significant beneficial health effects, particularly on CVD but many other diseases as well.

## 4. Cardiovascular

### 4.1 Animal Studies

Coronary artery disease is characterized by cholesterol rich blockages (plaques) in the artery that restrict blood flow to the heart (atherosclerosis). The decrease in energy supply to the heart due to the arterial plaques limits the ability of the heart to contract or, if severe enough, will induce the death of the heart cells in the part of the heart supplied blood flow by that coronary artery. This results in a myocardial infarction. The effect of dietary flaxseed on both the progression of coronary plaques and on the myocardial infarction itself has been studied in animal models.

Inclusion of ground flaxseed in the diet of both rabbit and mouse models of atherosclerosis slows the progression of developing atherosclerotic plaques [24, 25, 26, 27]. This anti-atherosclerotic action was present whether the plaque was induced by cholesterol-enriched diets [25, 26] or diets high in trans fat [27]. The progression of a developing plaque is but one aspect of atherosclerosis. Since most adults already have extensive plaque deposition in their coronary arteries, learning if a compound has the capacity to regress a plaque that is already established is just as important a therapeutic question to address. Indeed, dietary flaxseed has been shown to regress an established stable atherosclerotic plaque [28].

Supplementation of the diet with flaxseed induces an anti-inflammatory action. The anti-inflammatory action has been shown as a depression in the expression of molecules directly associated with inflammation including mac 3 M3/H84, interleukin-6 (IL-6) and vascular cell adhesion protein 1 (VCAM-1) [26] or specific oxylipins that are implicated in the inflammatory process [29]. The oxylipins implicated with these anti-inflammatory effects include 12, 13 dihydroxyoctadecenoic acid and 9,10 dihydroxyoctadecenoic acid [29].

The anti-atherosclerotic effects of dietary flaxseed have been attributed to the anti- inflammatory actions of a component of flaxseed—ALA. ALA has shown both anti- inflammatory action and anti-atherosclerotic effects consistent with the hypothesis that dietary flaxseed achieves its actions via its rich ALA content [27]. A recent study has shown that dietary ALA is preferentially incorporated into specific phospholipids in the cell which may play a role in the atherosclerotic potential and signaling activity within the tissue [30].

Cell proliferation within the vasculature is also thought to represent an important process in the growing atherosclerotic plaque [31, 32, 33]. Cell cycle proteins within the evolving plaque are activated and the atherogenic plaque develops into a pathogenic problem with clinical symptoms [31, 32, 33]. Dietary flaxseed has been shown to induce anti-proliferative actions within the vasculature through an inhibition of proliferative cell nuclear antigen (PCNA) expression [26]. Therefore, this represents an additional mechanism for the anti-atherosclerotic action of dietary flaxseed.

Flaxseed also contains SDG which is metabolized in the gut to form several enterolignans which can then gain access to the systemic circulation in the body [17, 34]. These enterolignans possess antioxidant capacity [35]. Because the oxidation of lipid moieties is thought to represent a key process in the atherosclerotic process [36], its inhibition by the enterolignans or by SDG has been suggested to represent a critical anti-atherogenic mechanism [35]. It is important to recognize that SDG does not enter the systemic circulation so it could not possibly generate a direct anti-atherogenic action [37]. However, the enterolignan metabolites of SDG can express phase 2 protein inducer which may be able to decrease oxidative stress [15]. Enterolignans themselves possess only weak anti-oxidative capacity so this direct action is unlikely to represent anything more than a supportive role in the anti-atherosclerotic effects of dietary flaxseed.

It is reasonable to expect that if flaxseed can prevent or slow the development of an atherosclerotic plaque in the artery, one would assume that the subsequent myocardial infarction would be prevented or the damage lessened. However, this hypothesis was tested even more directly by examining if supplementation of the diet with flaxseed prior to the induction of a myocardial infarction could occur even in the absence of any plaque development. Parikh and colleagues [37] found that dietary flaxseed reduced the size of a myocardial infarction by 20% when the infarct was produced artificially by tying off the coronary artery in the absence of atherosclerosis. Every 5% increase in infarct size is associated with a 20% increase in 1-year all-cause mortality or heart failure hospitalization. Clearly, a 20% reduction in infarct size will have significant implications for survival post infarct and decreases the incidence of heart failure and duration of hospitalization post infarct. Furthermore, in another animal model that exhibited spontaneous mini-infarcts in the heart, inclusion of milled flaxseed in the diet resulted in a significant reduction in the incidence of the myocardial infarcts [38]. Better cardiac contractile performance accompanied these reductions in cardiac damage [37, 38]. Dietary flaxseed has also been shown to reduce the incidence of arrhythmias during the reduction in flow to the heart [39] or after a myocardial infarction [37]. Arrhythmias are a central cause of heart failure after an infarction. Sudden cardiac death due to specific types of arrhythmias like ventricular tachycardia or fibrillation causes 25–50% death after a myocardial infarction. In addition, sudden cardiac death is 10-fold higher in the first 30 days after a myocardial infarction. Preventing arrhythmias immediately after an infarction will have significant beneficial protective effects on survival after the infarction. In summary, the ingestion of ground flaxseed will reduce myocardial damage either directly or by indirectly limiting the progression of arterial atherosclerosis. The reduction of infarct size and inhibiting arrhythmias is directly related to the improvement in heart function and duration of survival after a heart attack.

Data have been generated to determine the mechanism whereby dietary flaxseed can be cardioprotective during insults like ischemia and ischemic reperfusion [37, 38, 39]. The ALA content within flaxseed may exert a significant beneficial protection to the heart during these challenges. ALA can induce a modification of cell apoptosis as a mechanism of action. ALA inhibits the apoptotic pathway during ischemia/reperfusion [40]. It also prevents the formation of oxidized phospholipids during ischemia/reperfusion [40]. Oxidized phospholipid products that are generated during the ischemic/reperfusion insult have been proposed to induce cardiotoxicity through a ferroptotic mechanism [41, 42].

### 4.2 Human Trials

Nutraceuticals have been used successfully in clinical trials [43]. However, clinical trials using a nutraceutical or a nutritional supplement like flaxseed offer particular challenges in compliance, food delivery, food quality, food taste and acceptability, uncontrolled access to the trial food, as well as the use of an appropriate placebo, that drug trials do not have to overcome [44]. Despite these challenges, valuable data have been generated using flaxseed as a dietary intervention to determine its efficacy against CVD. These data have been generated in two CVD areas: cholesterol regulation and blood pressure control.

Concentrations of cholesterol circulating in the blood have been positively correlated with CVD. Elevated levels of plasma cholesterol are associated with a higher incidence of heart attacks and strokes [45]. The power of this association has been so strong as to initiate the development of a stable of pharmaceutical agents directly addressing the control of plasma cholesterol levels. The most commonly prescribed cholesterol-lowering drugs are statins. Supplementing the diet with flaxseed can reduce plasma cholesterol levels by 10–15% through a mechanism independent but complementary to statins [46]. Higher daily doses of ground flaxseed are required to achieve this effect (30–40 grams/day). In 2014, Health Canada was so persuaded by data on the cholesterol-lowering capacity of flaxseed that it approved a health claim linking the eating of ground whole flaxseed to blood cholesterol lowering, one of only eleven health claims approved in Canada. This cholesterol-lowering effect of dietary flaxseed is unlikely to be achieved through its SDG or ALA content [27] but instead is accomplished due to its rich fibre content [46].

Blood pressure (BP) is one of the most important cardiovascular parameters to monitor in order to prevent CVD [47, 48]. It becomes even more important because of its silent nature. Individuals are frequently unaware of the presence of even extremely high blood pressure levels and, therefore, do not enlist a physician’s help until it is too late and clinical symptoms of a heart attack or a stroke are evident [49].

Supplementing the diet with ground flaxseed produced a significant blood pressure lowering effect and this effect was present as early as one month after a daily ingestion of flaxseed and the blood pressure-lowering action was maintained during a full year of dietary supplementation with flaxseed [50]. A daily ingestion of 30 g ground flaxseed reduced blood pressure by 10–15 mmHg in systolic blood pressure and about 7 mmHg diastolic blood pressure [50]. This degree of decrease in blood pressure was estimated to reduce the incidence of heart attacks and strokes by about 50% [12, 50]. Both central [51] and peripheral systemic blood pressure [50] have been reduced by dietary flaxseed. The anti-hypertensive action of flaxseed was attributed to its rich content of the omega-3 fatty acid, alpha linolenic acid (ALA) [29, 50]. Importantly for those who do not experience hypertension, dietary supplementation with flaxseed did not reduce blood pressure in those with normal levels of blood pressure or induce hypotension [50]. A recent systematic review and meta-analysis of 33 randomized trials with 2427 participants that used flaxseed supplementation concluded flaxseed significantly reduced both diastolic and systolic blood pressures and was effective alongside routine anti-hypertensive medications [52]. They found that flaxseed supplementation had greater effects when the dietary intervention was >20 weeks, at a dosage of ≥30 g/day, in participants with a BMI 25–30 kg/$m^{2}$, and in patients with hypertension [52].

**Fig. 1.**
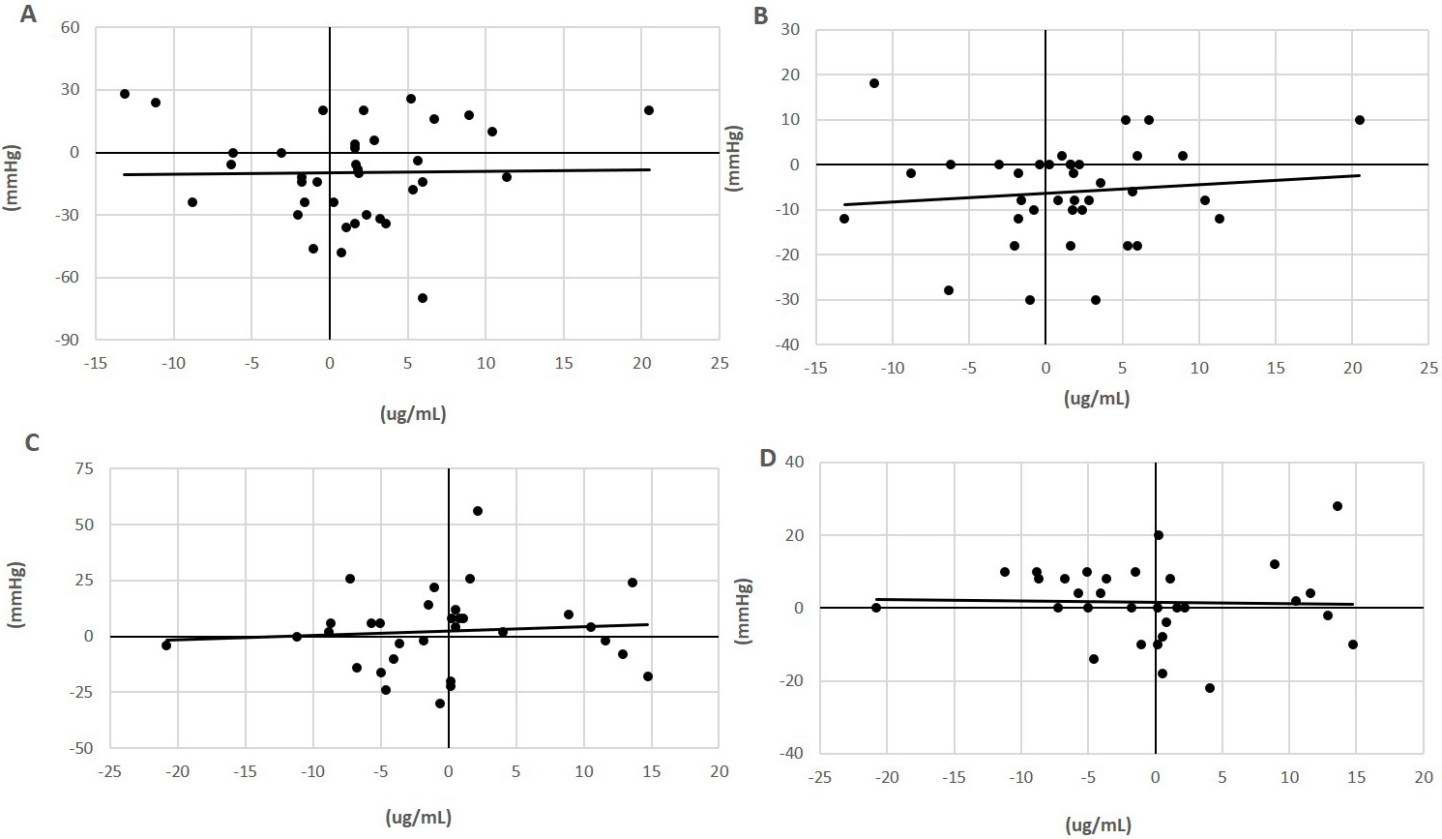
**Relationship of plasma L-arginine with blood pressure in flaxseed-supplemented subjects**. Plasma concentrations of L-arginine (ug/mL) in subjects with (milled flaxseed) (A,B) or without (control) (C,D) supplementation in the diet for 0 to 6 months and its correlation with the change in systolic (A,C) and diastolic blood pressure (mmHg) (B,D) [50]. The correlation of coefficient values were not statistically significant. L-arginine was assayed in plasma using the L-Arginine ELISA Kit from Immusmol (#IS-I-0400R) with a microplate reader set to 450 nm absorbance.

Flaxseed contains a relatively large concentration of L-arginine [15, 53]. L-arginine is thought to act as an important precursor to stimulate nitrogen oxidase synthase (NOS) activity [54]. It is logical, therefore, to hypothesize that the vasorelaxant effects exhibited by dietary flaxseed, particularly in the form of reduced BP values [50], maybe due to its delivery of L-arginine to the circulation. However, plasma samples collected from subjects ingesting a flaxseed supplemented diet (30 grams of ground seed/day for up to one year) exhibited no statistically significant increase in plasma L-arginine levels (Fig. 1). In addition, there was not a significant correlation of plasma L-arginine concentration with BP values (Fig. 1).

These data are in conflict with the rapid decrease in systolic blood pressure induced when spontaneously hypertensive rats were orally administered a protein isolate (200 mg/kg body wt.) obtained from flaxseed that contained a high amount of arginine [53]. The possibility exists, therefore, that the rich L-arginine content of flaxseed may contribute to the anti-hypertensive actions of flaxseed.

## 5. Cancer

Although cardiovascular disease has received considerable research attention recently, other diseases are influenced by the inclusion of flaxseed in the diet. The area that has received the most research attention involves cancers. Flaxseed has been shown in experimental studies to have a positive preventive action against breast [55, 56, 57], ovarian [58, 59], colon [60], brain [61], lung [62] and leukemia [60] cancers. Flaxseed can work in the presence of conventional chemotherapy to promote their activities or independently [56]. The mechanism of action appears to be achieved through a promotion of apoptosis [61], altering circulating sex hormone levels [59, 63], decreasing omental adiposity [58], and by reducing oncogenic microRNA [57] and proto-oncogene expression [61].

The component(s) within flaxseed that may be inducing these anti-oncogenic effects includes its lignan [56, 60], ALA [55], and/or DHA contents [55, 57]. It is important to recognize that although many anti-cancer drugs can reduce the progression and recurrence of certain cancers like breast cancer, they can unfortunately also induce heart disease that can progress to heart failure when the drugs are administered in large dosages or for prolonged periods [64]. The administration of flaxseed in the diet can prevent the cardiotoxic actions of breast cancer drugs like doxorubicin and trastuzumab without influencing the anti-oncotic actions of these drugs [65]. Dietary flaxseed was equivalent to conventional angiotensin-converting enzyme (ACE) inhibitors like perindopril that are conventional therapies to inhibit the cardiotoxic effects of breast cancer drugs [66]. Dietary flaxseed, therefore, has doubled its beneficial usage in cancer: firstly, as a therapy to prevent the progression of the tumour, and, secondly, as an inhibitor of the serious, life threatening side-effects of the cancer drugs on the heart.

Manipulating cell proliferation or cell death in an infected tissue are mechanisms whereby cancer can grow. The content of ALA within flaxseed may alter cell growth. ALA can be both pro- and anti-apoptotic depending upon the condition and/or tissue involved. For example, although ALA is anti-apoptotic in the heart [40], it is pro-apoptotic in tumours [67]. This agrees well with the cardioprotection ALA provides during ischemia and its anti-cancer effects.

## 6. Non-Alcoholic Fatty Liver Disease (NAFLD)

Non-alcoholic fatty liver disease (NAFLD) is now one of the most prevalent chronic liver diseases in the world [68]. Its incidence is ~25% across the globe [69] but this is probably only the tip of the iceberg which we will see in the coming decades as the co-morbidities for NAFLD (obesity and diabetes) are predicted to rise dramatically [70]. The risk factors identified for NAFLD include obesity, insulin resistance and hyperglycemia, hypertension and dyslipidemia (metabolic syndrome).

At present, a pharmacological therapy for NAFLD is unavailable. There is experimental and clinical evidence beginning to be published which suggest that dietary flaxseed may be an effective therapeutic option for those with NAFLD. In mice that are genetically lacking a protein to regulate blood cholesterol levels, a diet containing high amounts of fat and cholesterol will induce characteristics of NAFLD [71]. Dietary flaxseed oil administered even in the presence of this high fat diet improved indices of NAFLD [71]. It also attenuated biomarkers of inflammation, oxidative stress and lipid dysfunction [71]. In another study, a genetically unaltered mouse model fed a high fat diet to induce NAFLD again discovered that milled flaxseed provided a significant protective effect on liver health [72]. The study authors attributed this to a flaxseed-associated improvement in the gut flora- and microbiota-related bile acids [72]. Controlled studies of the effects of flaxseed ingestion in patients with NAFLD are few in number but the recent results are encouraging. In randomized, double blinded, controlled studies in patient populations with NAFLD, including flaxseed oil [73], milled flaxseed [74] or omega-3 fatty acids from flaxseed (ALA) or fish oils (eicosapentaenoic acid (EPA) or docosahexaenoic acid (DHA)) all improved the plasma lipid levels, liver histological profile and plasma indices of NAFLD [75]. These results show promise for the continued use and study of dietary supplementation with flaxseed oil and/or milled flaxseed for the treatment of NAFLD.

## 7. Diabetes

**Fig. 2.**
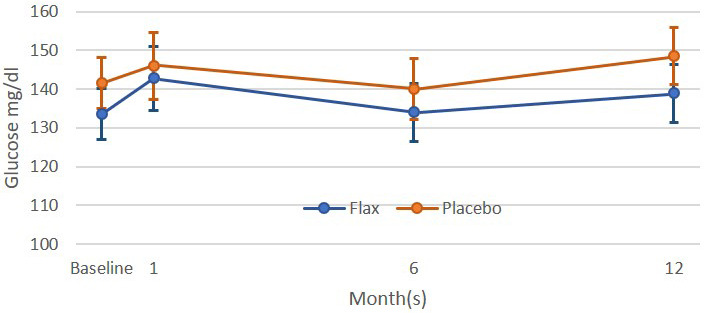
**Plasma glucose levels in patients with peripheral artery disease were subjected to a placebo or flaxseed supplemented diet for up to one year [50]**. All values were not statistically different (*p *> 0.05) from one another. Sample sizes were 52, 47, 41 and 41 for the 0, 1, 6 and 12 month time points, respectively, for the control group and 58, 52, 45 and 43 for 0, 1, 6 and 12 month time points, respectively, for the flaxseed supplemented group.

The incidence of diabetes is growing at an alarming rate across the world. The capacity for dietary flaxseed to impact on the incidence, severity, and/or control of diabetes would represent an important action. Supplementation of the diet with flaxseed has been reported to lower blood glucose levels in pre-diabetic patients [76] and in patients with Type II diabetes [77, 78]. Lignan extracts from flaxseed like SDG have also been reported to decrease blood glucose levels in Type II diabetics [79] and in animals with Type I diabetes [80]. However, it is important to recognize that patients with peripheral arterial disease (many of whom were in a state of well controlled diabetes through the use of a variety of glucose-lowering therapies) exhibited no significant change in plasma glucose levels when their diet was supplemented for up to one year with ground flaxseed (Fig. 2, Ref. [50]). The direct involvement of SDG in the lowering of blood glucose levels, as suggested elsewhere [79, 80], is doubtful when SDG is restricted from entering the systemic circulation.

## 8. The Gut Microbiome

The investigation of the flora in the gut has become an increasingly important area of research due to the effects this organ has on the function of other parts of the body. Flaxseed supplementation to the diet of lean and obese rats improved the gut microbiota by decreasing the abundance of *Blautia* and *Eubacterium dolichum * [81]. Flaxseed supplementation induced significant changes in gut bacterial diversity even in the presence of a high fat, high sucrose diet [81]. The levels of the bacterial phylum *Bacteroidetes* and of the phylum *Firmicutes* were influenced by dietary flaxseed in directions that would enhance gut health [81]. Using aging rats, supplementation of the diet with flaxseed increased the presence of four families of *Lactobacillus* and six families of *Clostridium*, as well as the relative abundance of the genera *Ruminococcaceae* UCG-005 and *Prevotella* 9, bacteria associated with aging [82].

From a purely functional standpoint in the intestines, dietary flaxseed generates a significant effect on constipation. Daily doses of flaxseed can relieve symptoms of both mild [83] and chronic constipation [84]. The efficacious dose varied from 6 to 50 g/day [83, 84]. Whole flaxseed [85], ground flaxseed [83, 84, 86], partially defatted flaxseed [87], and flax oil [86] were all successful in reducing symptoms of constipation. Surprisingly, flaxseed oil also demonstrated beneficial effects against diarrhea [86]. The anti-constipation effects were evident in elderly populations [83]. Flaxseed was superior in its actions against constipation than more traditional therapies like psyllium [77] or lactulose [84].

The functional effects of flaxseed on the gastrointestinal system have also been tested in select populations with co-morbidities. The pro-defecation actions of a dietary supplementation with flaxseed were observed in a patient population with Type 2 diabetes [77]. Patients who underwent chemoradiation for lung cancer, however, did not tolerate flaxseed supplementation to their diets very well [62]. Patients with irritable bowel syndrome (IBS) found that supplementation of the diet with flaxseed in either whole or ground form provided significant relief from symptoms of IBS [85].

In summary, flaxseed enriches the bacterial diversity within the gut which can have a positive impact upon the health and performance of a variety of bodily functions outside of the gastrointestinal system. Supplementation of the diet with flaxseed in a variety of forms and incorporated within many different foods can provide a very effective relief from constipation which can present itself with normal aging or when a variety of co-morbidity situations become evident. Flaxseed is also capable of replacing conventional drug therapies which may concomitantly reduce side effects commonly observed when employing these medicinal therapies.

## 9. Menopausal Symptoms

Flaxseed contains SDG which is metabolized in the intestines to form the enterolignans, enterodiol (END) and enterolactone (ENL) [17]. SDG does not gain access to the circulation but END and ENL do circulate and they possess biological action. Flaxseed is not a usual component of the diet today for the general population. As a result, the baseline enterolignan concentrations in plasma typically fall well below the limit of detection for END and ENL in a healthy, control population [17]. Total enterolignan (END + ENL) concentrations at baseline are low as well but detectable (15 nM). After 4-weeks of consuming muffins containing 30 g of milled flaxseed, subjects displayed average plasma END and ENL concentrations of over 200 nM for each enterolignans [34]. The ratio of END to ENL was 0.81. Total plasma enterolignans averaged over 500 nM after the flaxseed intervention period [34]. The age of the subject (18–69 years of age) had no effect on the capacity to metabolize SDG into enterolignans [34]. Ethnic variation, however, has been suggested to have an effect on the gut’s capacity to metabolize SDG in flaxseed [88].

The enterolignans possess weak estrogenic action and may have some depressive action against menopausal symptoms [89]. Because of this, it was a logical next step to test the capacity of dietary flaxseed and its metabolites to alter female hormone levels and menopausal symptoms. Women consuming flaxseed increased the ratio of serum 2- hydroxyestrone and 2:16 alpha hydroxyestrone levels [63]. These changes in sex hormone levels were suggested to have implications for breast cancer [63] and potentially menopausal symptoms. However, in a major trial where menopausal women consumed 40 g/day for 1 year, no significant change in menopausal symptoms was detected [89].

## 10. Bone Density and Strength 

Dietary flaxseed does not appear to induce significant changes in bone composition or strength in healthy adults. Forty grams of flaxseed per day for 12 months resulted in no significant change in bone mineral density in menopausal women [89]. However, flaxseed oil [90, 91] or ground flaxseed [92] have significant beneficial effects on bone composition, mineral density and content, area, breaking strength and maximal force in young developing rats [90, 91, 92, 93]. This is likely due to the ALA and omega-3 fatty acid content in flaxseed. Even delivering the flaxseed to the mothers during lactation was sufficient to induce these positive effects on bone development in the weaning pups [90, 92]. Furthermore, in hemodialysis patients where bone disorders are common, augmentation of the diet with flaxseed oil reduced bone resorption in these patients [94]. It is possible, therefore, to make preliminary conclusions based upon the limited amount of data available in the literature. Although flaxseed supplementation is without significant impact on bone health during normal adult life, it is very important for bone health during early postnatal development and during stressful conditions where bone composition and strength parameters may be compromised. However, additional research in these areas would be helpful to unequivocally support this conclusion.

## 11. Wound Healing

Flax dressings have been in use to promote the healing of wounds for thousands of years. In ancient Mesopotamia, Egypt, Greece and the Roman Republic, their armies used linen dressings for wounds incurred in wartime [95]. These were used in Rome during gladiator times when wounds were a frequent result of conflicts in the stadiums of the time [95]. More recent research studies have carefully evaluated the efficacy of linen dressings to accelerate the healing process. Generally, the studies have proven the efficacy of using linen or flax oil to promote wound healing [95, 96, 97, 98, 99, 100, 101]. There exist some conflicting studies that have not shown much beneficial effect [102, 103] and a concern of specific preparations of linen that may induce dermal irritation [99]. However, the majority of evidence would support the continued use of linen in promoting wound healing. The mechanisms by which flax fibres induce a protective action include an inhibition of skin cell apoptosis, promotion of skin cell growth [96], inhibition of inflammation [95, 98], increased collagen synthesis and fibroblast proliferation [98], and protection against oxidative damage [95]. Combinations of flax extracts in the fibres with additional complementary wound healing elements appear to accelerate the wound healing process even further [102, 104, 105].

## 12. Functional Exercise Capacity

There are a limited number of studies which have examined the effects of dietary flaxseed on the ability of subjects to exercise. Patients with peripheral arterial disease (PAD) have blockages in their extremities that severely limit their capacity for physical exercise. This can limit their ability to walk, for example, for longer than 60 seconds without incurring severe ischemic pain [106]. Supplementation of the diet with flaxseed can improve aspects of vascular function [25, 50] and this may give cause to believe that this intervention may have a positive effect on the ability of the PAD patients to perform physical exercise. The inclusion of 30 g ground flaxseed daily into the diet of patients with PAD for up to one year, however, did not improve absolute claudication time (total duration of exercise) or relative claudication time (time to onset of ischemic pain with exercise) [106].

## 13. General Mechanisms of Action of Flaxseed

**Fig. 3.**
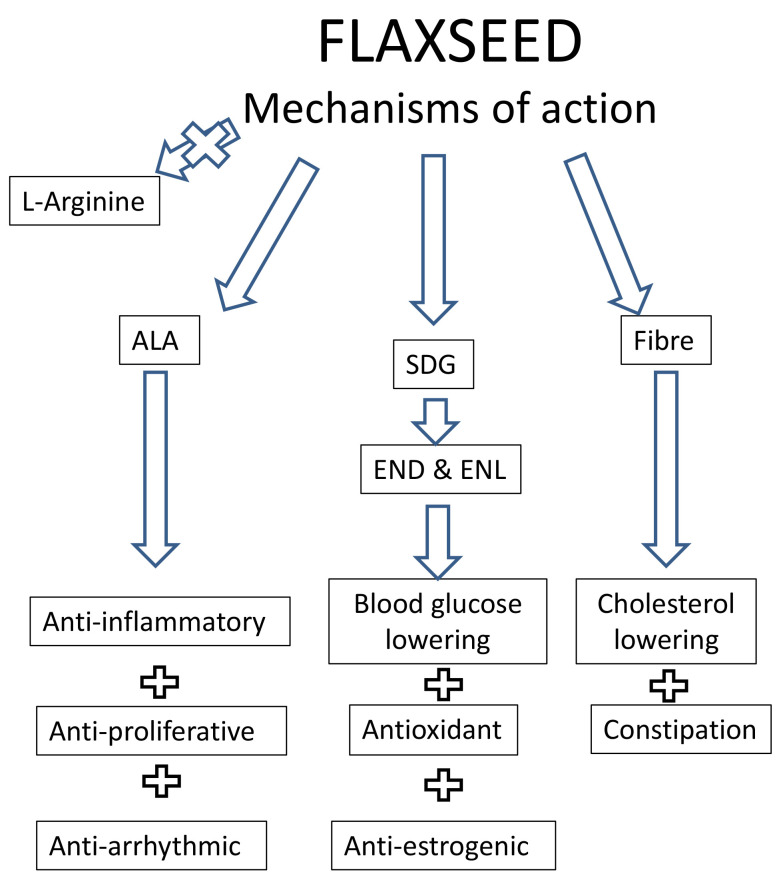
**A summary of the proposed mechanisms of biological action of dietary flaxseed**. Abbreviations: ALA, alpha linolenic acid; SDG, secoisolariciresinol diglucoside; END, enterodiol; ENL, enterolactone.

One way to convince the public and the scientific community of the veracity of the findings in a lab is to identify the mechanism by which a compound produces a physiological effect. This provides some confidence in the results and an explanation for why a functional food or its nutraceutical compound elicits the effects it does *in vivo* or* in vitro*. The effects of flaxseed have been associated with a variety of target pathways and proteins through which the actions shown in studies discussed above have been achieved. The various mechanisms proposed to account for the biological actions of dietary flaxseed have been discussed in each of the sections of the text above as they relate to specific biological activities of flaxseed. These are summarized in Fig. 3 below:

Some of the biological effects of dietary flaxseed may be excluded as having a significant action. For example, milled flaxseed delivered even at higher doses to both healthy participants or a patient population did not induce any changes in platelet aggregation induced by collagen or thrombin [18, 50]. However, flaxseed oil at higher doses can reduce platelet activity [54].

## 14. Age and Sex Considerations

Age and sex are important considerations in ischaemic heart disease and many other pathologies [107]. This may have implications for the effects of dietary interventions like flaxseed.

### 14.1 Age

It is entirely plausible that the absorbance of flaxseed bioactives from the gut may be influenced by an advancing age [108]. However, this was not the case for both ALA and the enterolignans. Subject age (18–29 years of age in comparison to 45–69 years old) does not that will cause

to be a determining factor in influencing ALA absorption from a flaxseed supplemented diet [108]. The metabolism of longer chain fatty acids like EPA from ALA was also similar in both age groups. The metabolism of flaxseed into triglycerides, however, was lower in the younger age group in comparison to the older subjects.

Whereas ALA absorption and metabolism from a flaxseed supplemented diet was unaffected by the age of the subjects, the plasma availability of enterodiol (END), enterolactone (ENL) and total enterolignans after 4 weeks of flaxseed supplementation was altered by the age of the subject (18–29 years of age in comparison to 45–69 years old) [34]. Although total enterolignans levels were increased in both age groups, there was a smaller conversion of the SDG in flaxseed to ENL in younger subjects than in the older subjects [34].

The effect of dietary flaxseed on inflammatory lipids in younger (19–28 years old) and older (45–64 years of age) subjects has also created interesting results [109]. At baseline, pro-inflammatory oxylipins were significantly greater in older compared to younger subjects. However, after 4 weeks of flaxseed consumption, these circulating pro-inflammatory oxylipins were reduced to levels approximating those detected in the younger group [109]. The authors concluded that dietary flaxseed may have the capacity to disrupt the aging process in a beneficial manner by reducing the concentrations of pro-inflammatory molecules in circulation in elderly subjects.

### 14.2 Sex 

The most comprehensive analysis of the influence of sex on the biological effects of a flaxseed enriched diet was carried out in JCR:LA-cp rats. Most of the lipid responses in the circulation to the supplementation of flaxseed in the diet were similar in male and female animals [38]. The increase in the frequency of ischemic lesions in response to a high fat, high sucrose diet was similar in male and female rats and a significant reduction by including flaxseed in the diet was similar again in both sexes [38]. This sexual similarity did not extend entirely to the gut microbiota. Genetic obesity affected the richness and the alpha diversity of the gut microbiota in female rats alone [81]. However, flaxseed supplementation to the diet was beneficial to the gut microbiota in both male and female animals [81].

## 15. How do We Include Flaxseed in the Diet?

This section will briefly summarize the salient features of what we currently know is important about the inclusion of flaxseed in the diet.

### 15.1 Stability of Flaxseed 

Whole flaxseed is stable for years when stored at room temperature. Baking at high temperatures does not affect the oil composition or integrity [110]. Crushing, milling or grinding the hard exterior coat of the seed, however, exposes the inner contents to oxidation. Despite this vulnerability to oxidation, milled flaxseed can still be stored at –20 °C for years without evidence of the generation of oxidation products within the seed [111]. Milled flaxseed can even be stored at room temperature for years if the container is properly and securely sealed [110]. Again, baking milled flaxseed into breads, rolls, buns, tea biscuits and pasta does not result in changes in the content of oxidized lipids or SDG [110].

### 15.2 The Recommended Daily Dosage of Flaxseed 

There is no recommended dose of flaxseed that should or could be ingested daily. Sustained delivery of milled flaxseed at concentrations up to 50 g/day has been reported [89]. In healthy, younger adults, 10 g/day of milled flaxseed was sufficient to significantly increase plasma levels of ALA and total enterolignans [18]. A dosage of 30 g flaxseed/day baked into muffins was necessary to induce the conversion of ALA into EPA in blood [18]. Thirty grams/day of milled flaxseed induced positive changes in blood pressure in hypertensive individuals [50]. A comprehensive analysis of the lowest effective dose of milled flaxseed that can achieve decreases in blood pressure has not been reported.

### 15.3 The Optimal Form of Flaxseed to Ingest 

Oral ingestion of similar dosages of ALA delivered in whole seed, milled seed or flax oil resulted in very different plasma ALA levels. Whole seed did not produce a significant elevation in plasma levels of ALA whereas milled seed and flax oil did [13]. The age of the person did not influence bioavailability of the bioactive [108]. The food into which flaxseed was incorporated did influence the acceptability of the food. Milled flaxseed was incorporated into different flavours of muffins, bagels and snack bars as well as tea biscuits, pasta and buns. The participants in the trial were then free to choose each day which foods they would like to consume over the course of a full year. The bagels were consumed the most followed by the muffins, snack bars, biscuits, pasta and buns [112]. The more flavourful the additive to the functional food, the more it was chosen [112]. This trial was completed in Canada and the ethnic origin and country of the population under study would be expected to influence these results. Importantly, these foods were well tolerated and the participants ate them for the full year. Few complications (i.e., bloating, cramping, gas) were noted [50]. However, if the subject is not accustomed to a high fiber content in their diet, this will induce all of these concerns soon after ingestion. A gradual introduction of flaxseed into the diet is recommended to avoid the bloating and gaseous reactions that will cause compliancy issues with continuing the dietary supplementation.

“Milk” beverages containing milled flaxseed show great promise to increase the consumption of flaxseed. Beverages that contain oat, soy, cashews and hempseed are now common in the marketplace. These “milk” beverages have also used flaxseed as a convenient method by which to achieve a daily dose of milled flaxseed. However, although some flaxseed beverages have appeared in the marketplace that contain milled flaxseed, the flaxseed does not remain in suspension like other vegetarian “beverages” obtained from oat or cashews or soy. The flaxseed drops to the bottom of the container and is less compatible with the daily diet in terms of visceral acceptability and a consistent dosage. However, in a recent development, the Manitoba Flax Milling Corporation has achieved a flaxseed product that is so finely milled through a special patented process that the flaxseed remains in suspension and does not descend to accumulate in the bottom of the container. This product should expand the opportunity for the public to include flaxseed in their daily diet.

## 16. Conclusions 

The inclusion of flaxseed in the diet has a great number of potential benefits for the health of both elderly and young adults. In the past, it has been difficult to find suitable foods in the marketplace that contain flaxseed in doses that would have an impact upon our health. These barriers to the consumption of flaxseed are slowly disappearing. With an increase in the number and quality of studies focused on the physiological and pathophysiological effects of dietary flaxseed, our knowledge concerning the rationale for the inclusion of flaxseed in our diet has become sounder and more convincing. Currently available data strongly encourage further work in this area to replicate and advance our knowledge of the biological actions of dietary flaxseed. There is still much to be learned about the use of flaxseed in the diet, as discussed in detail elsewhere [113]. The lowest effective dosage of flaxseed, additional information on its biological mechanisms of action, how to incorporate it more easily into more food products, the effects on the brain and the economic impact of using flaxseed (both on the health side of the ledger and the agricultural side) are just a few of the areas that require further study.
